# Association Between Malnutrition and Food Texture Levels in Integrated Facilities for Medical and Long-Term Care

**DOI:** 10.7759/cureus.61929

**Published:** 2024-06-08

**Authors:** Yoji Kokura, Akio Shimizu

**Affiliations:** 1 Department of Nutritional Management, Keiju Hatogaoka Integrated Facility for Medical and Long-Term Care, Hosu, JPN; 2 Department of Food and Health Science, Faculty of Health and Human Development, The University of Nagano, Nagano, JPN

**Keywords:** insurance, prevalence, long-term care facility, food quality, deglutition disorders

## Abstract

Objectives:* *This study aims to investigate the association between malnutrition using the global consensus criteria and food texture levels in residents of Integrated Facilities for Medical and Long-Term Care (IFMLCs), which are new long-term care insurance facilities in Japan.

Methods: This single-center study had a retrospective cross-sectional design. The study was conducted from November 1 to 30, 2021, and the study participants were residents admitted to an IFMLC during the study period. Malnutrition was diagnosed according to the Global Leadership Initiative on Malnutrition (GLIM) criteria. Food texture levels consumed by patients at admission were categorized based on the International Dysphagia Diet Standardization Initiative (IDDSI) framework. Multivariate logistic regression models were used to determine the association between the food texture levels consumed and malnutrition.

Results: A total of 98 older residents were analyzed in this study. The median age of the participants was 88 years, and 68 (69%) female participants were included. The IDDSI framework levels were 24% in levels 7 and 6 and 26% in levels 5 and 4. A significant difference in the prevalence of low BMI, reduced muscle mass, and reduced food intake or assimilation was noted between IDDSI framework levels 4 and 7. Multivariate logistic regression analysis was performed for malnutrition, adjusting simultaneously for potential confounders. IDDSI level 4 (odds ratio, 5.074; 95% confidence interval, 1.059-28.092; p=0.042) consumption was independently associated with malnutrition.

Conclusions: The consumption of lower food texture levels categorized using the IDDSI framework was associated with a higher malnutrition prevalence in IFMLC residents.

## Introduction

Malnutrition in long-term care facilities (LTCFs) is a significant concern for healthcare professionals. Malnutrition is observed in 1.5-66.5% of older individuals admitted to LTCFs and nursing homes [1,2]. Malnutrition is regularly linked with cognitive impairment, difficulties swallowing, functional decline, and depression [2]. However, malnutrition definitions are variable, and a consistent definition is needed to truly tackle the issue of malnutrition in the nursing home setting [2]. The Global Leadership Initiative on Malnutrition (GLIM) criteria [3] target the priority of adopting global consensus criteria by several of the major global clinical nutrition societies so that malnutrition prevalence, interventions, and outcomes may be compared worldwide. Therefore, in LTCFs, performing nutritional diagnosis using the GLIM criteria and investigating malnutrition-related factors are important.

Japan, the country with the oldest population in the world, is experiencing an escalating demand for long-term care. In response, the Japanese public long-term care insurance system introduced the Integrated Facilities for Medical and Long-term Care (IFMLCs) in 2018, which offers extensive medical, chronic, and nursing care services. By 2020, 515 IFMLCs were providing 32,634 beds designed to support the requirements of older adults in need of nursing care [4]. Among the older residents in IFMLCs, 29% are estimated to be malnourished according to the GLIM criteria, a rate that could be comparable to or higher than that found in traditional nursing homes [4]. The connection between malnutrition, as evaluated using the GLIM criteria, and the use of texture-modified diets (TMDs) in IFMLCs remain unclear. Dysphagia prevalence in LTCFs and nursing homes is estimated to range from 12.8% to 52.7% [5-7]. To treat patients with oropharyngeal dysphagia, TMDs and fluid modifications are used [8]. However, it has also been reported that TMDs offer lower energy, protein intake, and nutrient amounts than a regular diet [9-11]. Therefore, in LTCFs with several older residents and a high incidence of dysphagia, TMDs may be associated with a potential risk of malnutrition. However, as IFMLCs are a novel type of LTCF, no studies have investigated the relationship between malnutrition assessed using the GLIM criteria and TMDs.

Identifying the association between malnutrition assessed using the GLIM criteria and food texture levels could provide insights into strategies for preventing or treating these conditions. Therefore, this research sought to explore the association between malnutrition using the global consensus criteria and food texture levels in residents of IFMLCs, which are new long-term care insurance facilities in Japan.

## Materials and methods

Participants

**Figure 1:**
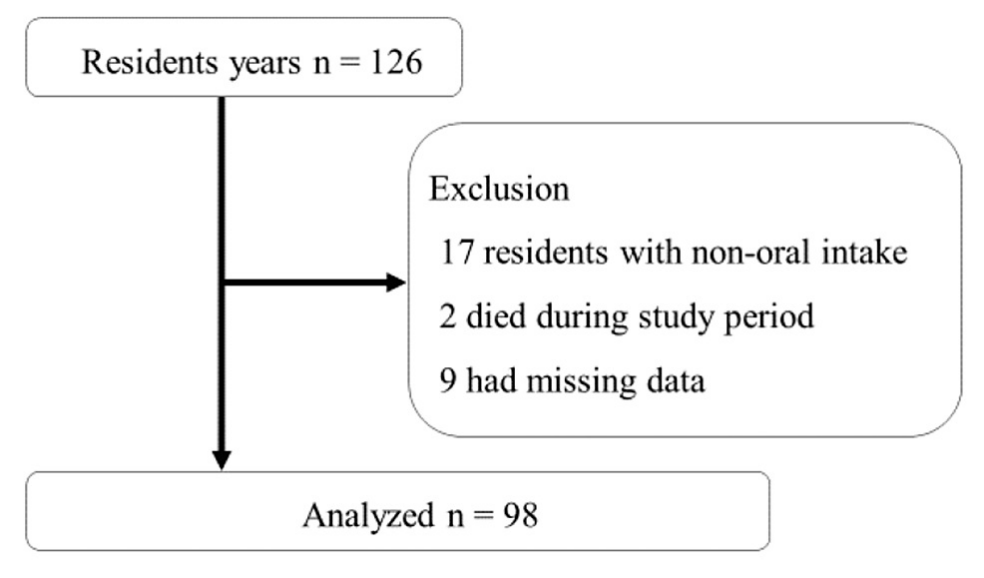
Ninety-eight residents are analyzed in the study Image Credit: Author

This study was carried out at a single center using a cross-sectional design. The Keiju Hatogawaka IFMLC in Ishikawa, Japan, was the facility involved in the study. The IFMLC offers rehabilitation and nutritional management services in accordance with the Japanese insurance system [4]. The research was conducted from November 1 to 30, 2021, with participants being residents admitted to the IFMLC during this time. Clinical data were collected within the study timeframe. Residents who had missing data or died during this period were excluded from the study. Moreover, residents with nonoral intake (only enteral or parenteral nutrition) were excluded. Ultimately, 98 of 126 residents were analyzed, excluding 17, 9, and 2 residents with nonoral intake, missing data, and who died, respectively (Figure 1). The study was approved by the Tosenkai Nursing Facility Ethics Committee (approval number: ID 2023-01). Due to its retrospective research, it was not feasible to obtain written informed consent; consequently, the requirement for written informed consent was not waived. Instead, participants were notified of their right to opt out of the study through announcements posted on the IFMLC bulletin board and website.

IFMLC

Providing comprehensive care management is the fundamental policy of IFMLCs. The services at these facilities encompass medical care, nursing, functional training, and everyday living assistance for individuals in need of long-term medical care, all guided by the facility's service plan [4]. Various treatments, such as medications and diagnostic tests, are provided after consulting with a physician in IFMLCs. Additionally, end-of-life care is offered. Notably, the delivery of these medical services distinguishes IFMLCs from other LTCFs. The interdisciplinary team at IFMLCs includes registered dietitians, occupational therapists, physical therapists, pharmacists, nurses, caregivers, care managers, and physicians. Nutrition care management is a Japanese long-term care insurance nutrition management method [4]. Each plays a vital role in the multidisciplinary approach to care. The dietitian oversees all aspects of nutritional care based on the nutritional care strategy.

Parameters

Clinical data were systematically collected as part of routine evaluations. For the present study, pertinent information was extracted from the medical records. All clinical data were gathered during the duration of the study. This included the demographic details of the participants, such as age and sex, their status prior to IFMLC admission, the length of stay at the IFMLC, the main reasons for admission to the facility, nursing care level [12], comorbidities, weight loss before admission to the hospital, overall physical functionality, swallowing capabilities, activities of daily living (ADLs), and the amount of rehabilitation received daily during the study period. The impact of comorbid conditions was assessed using the Charlson Comorbidity Index (CCI) score [13]. The Barthel Index (BI) was utilized to evaluate ADLs [14], with its 10-item total score ranging from 0 to 100, where higher scores reflect complete independence and lower scores suggest reduced physical function. Swallowing capability was assessed by the registered dietitian through the food intake level scale (FILS) [15], which is a subjective scale ranging from 1 to 10, where higher scores indicate better swallowing ability. Levels 1-3, 4-6, and 7-10 correspond to "no oral intake," "oral intake and alternative nutrition," and "exclusive oral intake," respectively. The visual estimation method [16], which is commonly employed in hospitals and care facilities to estimate food intake from plate waste, was used for assessing energy and protein intake. The registered dietitian calculated these intakes from the diet records provided by ward nurses and caregivers, considering both the energy and protein content of the provided meals. In addition, any enteral and parenteral nutrition was recorded, contributing to the total energy and protein intake data.

The International Dysphagia Diet Standardization Initiative (IDDSI) framework

**Figure 2:**
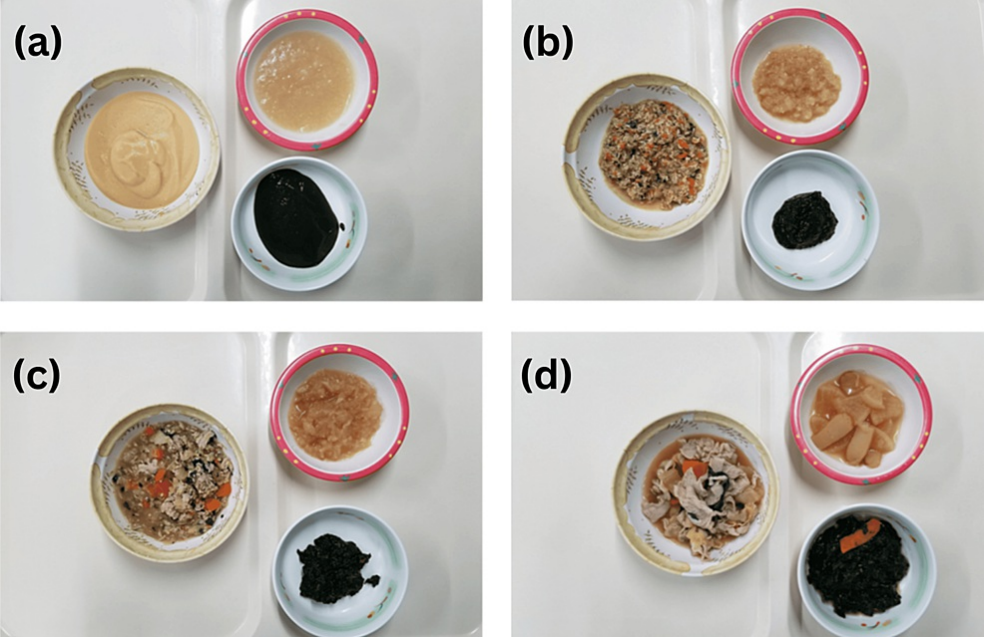
Meal forms at the Keiju Hatogawaka IFMLC (a–b) TMDs in the study: (a) level 4, pureed; (b) level 5, minced and moist; (c) level 6, soft and bite-sized; and (d) level 7, regular/easy to chew IFMLC: Integrated Facilities for Medical and Long-term Care, TMDs: texture-modified diets Image Credit: Author

Upon admission, the food texture levels consumed by participants were classified based on the IDDSI framework. This framework provides a universally standardized description of TMDs and liquids [17-19]. Registered dietitians assigned food texture levels following the guidelines described on the IDDSI’s official website (www.iddsi.org). These classifications included level 3, liquidized; level 4, pureed; level 5, minced and moist; level 6, soft and bite-sized; and level 7, regular or easy to chew (Figure 2) [17-19].

Nutritional status

A two-step approach for diagnosing malnutrition according to the GLIM criteria [3] was selected, involving first a screening to identify "at-risk" status using any validated screening tool, followed by an assessment to diagnose malnutrition. The Malnutrition Universal Screening Tool (MUST) was employed to assess malnutrition risk [20]. Scores of 0, 1, and 2 on the MUST correspond to low, medium, and high malnutrition risks, respectively [20]. A MUST score of 1 was thought to be suggestive of malnutrition risk in this research. The MUST is recommended as a screening tool in the GLIM criteria [3]. The phenotypic criteria for malnutrition include unintentional weight loss, a low BMI, and diminished muscle mass [3]. Specifically, these criteria are defined as a weight loss of ≥5% within the last six months or ≥10% beyond six months, a BMI of less than 20.0 kg/m2 for patients aged ≥70 years and 18.5 kg/m2 for those under 70, and a reduction in muscle mass [3]. The threshold for calf circumference used in diagnosing sarcopenia in Asian populations is less than 34 cm for males and less than 33 cm for females, as per the established criteria [21]. Anthropometric measurements were taken by registered dietitians on the nondominant or nonparalyzed limbs of participants while they were lying in a supine position. The two etiological criteria considered are a decrease in food intake or digestive/absorptive efficiency and disease burden or inflammatory conditions [3]. The specific etiological factors assessed included reduced food intake for a week or any decrease lasting more than two weeks, problems with assimilation such as dysphagia, vomiting, and diarrhea, and conditions contributing to disease burden or inflammation, such as congestive heart failure, chronic obstructive pulmonary disease, chronic kidney disease, and cancer [3]. A diagnosis of malnutrition is made when one or more of the identified phenotypic or etiologic criteria are present [3].

Statistical analysis

The statistical analysis was conducted using JMP 11.2.1 software (SAS Japan, Tokyo, Japan). Categorical variables were reported as numbers (percentages), parametric variables as means ± standard deviations, and nonparametric variables as medians (interquartile ranges). To analyze the differences in background characteristics across IDDSI levels, the χ2 test, Fisher’s exact test, one-way analysis of variance, and Kruskal-Wallis test were utilized. The Bonferroni correction and the Steel-Dwass test were applied for post hoc analyses. The Cochran-Armitage trend test was used to assess the prevalence of malnutrition across different food texture levels. Multivariate logistic regression models were employed to explore the relationship between food texture levels and malnutrition. Covariates for adjusting bias were chosen based on basic demographic factors, such as age and sex, primary diseases for IFMLC admission, the CCI, and variables previously identified in IFMLC population studies as influencing malnutrition, including ADLs (BI) [4]. The propensity score, summarizing all covariates included in its model, was used as a covariate in a regression model to analyze the effects of treatments [22]. Initially, logistic regression was conducted using the presence of TMDs, as defined by IDDSI framework levels of ≤5, as the dependent variable and each covariate as an independent variable, from which the propensity score was derived. Subsequently, multivariate logistic regression was performed using the propensity score and IDDSI levels as independent variables and the presence of malnutrition defined by GLIM criteria as the dependent variable. Adjustments were made for covariates based on the propensity score. A p-value of less than 0.05 was deemed to indicate statistical significance.

## Results

The median age of the participants was 88 (84-93) years, and 68 (69%) female participants were included. Of the participants, 30 (31%) were diagnosed with malnutrition according to the GLIM criteria. The baseline characteristics and statistical analysis of the participants among the IDDSI framework levels are shown in Table 1. The IDDSI framework levels indicated that 24 (24%) of participants were assessed at levels 7 and 6, and 25 (26%) were assessed at levels 5 and 4. In the comparison of baseline characteristics between participants with IDDSI framework levels, those with low IDDSI framework levels had higher nursing care levels, FILS and BI points, and an estimated time of rehabilitation dose (p<0.05). However, no differences in other clinical variables were observed between the IDDSI framework levels (Table 2).

**Table 1:** Baseline characteristics and statistical analysis of the participants among IDDSI framework levels Data are presented as medians (IQRs) or numbers (%). ^a)^Mann–Whitney U test, ^b)^Fisher’s exact test, ^†^p<0.001, level 7 versus level 4 using the post hoc Steel–Dwass test, ^¶^p<0.001, level 7 versus levels 4, 5, and 6 using the post hoc Steel–Dwass test, ^§^p<0.01, level 7 versus levels 4 and 5 using the post hoc Steel–Dwass test,^ ‡^p<0.05, level 7 versus level 4 using the post hoc Steel–Dwass test IDDSI: International Dysphagia Diet Standardization Initiative, IFMLC: Integrated Facilities for Medical and Long-term Care, CCI: Charlson Comorbidity Index, IBW: ideal body weight, IQRs: interquartile ranges

Characteristic	IDDSI framework levels				
	Level 7	Level 6	Level 5	Level 4	p-value
Number of individuals, n (%)	24 (24%)	24 (24%)	25 (26%)	25 (26%)	
Age	88 (84-91)	91 (84-96)	89 (81-93)	88 (84-92)	0.592 ^a)^
Female	14 (58)	15 (63)	18 (72)	21 (84)	0.207 ^b)^
Spent at IFMLC (days)	226 (171-674)	501 (166-891)	429 (279-1,269)	611 (382-1,274)	0.150 ^a)^
Nursing care level	2.5 (2-3) ^†^	3 (1-4)	4 (2.5-4.5)	5 (4-5)	<0.001 ^a)^
Primary diseases associated with IFMLC admission					0.107 ^b)^
Cerebrovascular disease	9 (37)	8 (34)	13 (52)	13 (52)	
Dementia	6 (25)	5 (21)	11 (44)	5 (20)	
Orthopedic diseases	2 (8)	3 (12)	0 (0)	4 (16)	
Heart failure	4 (17)	3 (12)	1 (4)	1 (4)	
Other diseases	3 (13)	5 (21)	0 (0)	2 (8)	
CCI (points)	2 (1-3)	2 (1-3)	2 (2-3)	2 (2-3)	0.195 ^a)^
Food intake level scale, median (IQR)	10 (9-10) ^¶^	8 (8-8)	7 (7-7)	7 (7-7)	<0.001 ^a)^
BI (points)	63 (43-75) ^§^	35 (15-69)	25 (10-53)	10 (0-18)	<0.001 ^a)^
Estimated time of rehabilitation dose (minute/day)	11 (10-11) ^‡^	10 (9-11)	11 (9-11)	9 (9-11)	0.005 ^a)^

**Table 2:** Nutritional characteristics based on GLIM components and nutritional intake in residents among IDDSI framework levels Data are expressed as medians (IQRs) or numbers (%). ^a)^Fisher’s exact test, ^b)^Mann–Whitney U test, ^†^p<0.05, level 7 versus levels 4 and 5 using the post hoc Steel-Dwass test, ^¶^p<0.01, level 7 versus levels 4 and 5 using the post hoc Steel-Dwass test, ^§^p<0.05, level 7 versus levels 4 and 5 using the post hoc Steel–Dwass test IDDSI: International Dysphagia Diet Standardization Initiative, MUST: Malnutrition Universal Screening Tool, BMI: body mass index, GLIM: Global Leadership Initiative on Malnutrition, IQRs: interquartile ranges

Characteristic	IDDSI framework levels				
	Level 7	Level 6	Level 5	Level 4	p-value
Number of individuals, n (%)	24 (24%)	24 (24%)	25 (26%)	25 (26%)	
MUST 1	14 (58)	21 (87)	22 (88)	22 (88)	<0.032 ^a)^
Phenotypic criteria					
Weight loss, presence, n (%)	6 (25)	6 (25)	6 (24)	7 (28)	1.000 ^a)^
Weight change (%), median (IQR)	−1.8 (−5.6-2.4]	−2.8 (−6.0-2.3)	−1.1 (−5.4-3.4)	−2.9 (−6.3-1.2)	0.857 ^b)^
Low BMI using the GLIM, presence, n (%)	9 (38)	17 (71)	20 (80)	19 (76)	0.009 ^a)^
BMI (kg/m^2^), median (IQR)	22 (17–24) ^†^	19 (17–21)	18 (16–20)	16 (14–19)	0.001 ^b)^
Reduced muscle mass, presence, n (%)	21 (87)	24 (100)	25 (100)	25 (100)	0.027 ^a)^
CC (cm), median (IQR)	30 (27-32)^ ¶^	27 (25-30)	25 (23-27)	23 (21-25)	<0.001 ^b)^
Etiologic criteria, presence, n (%)					
Reduced food intake or assimilation	1 (4)	0 (0)	0 (0)	4 (16)	0.044 ^a)^
Disease burden/inflammation	5 (21)	6 (25)	6 (24)	11 (44)	0.284 ^a)^
Energy intake (kcal/IBW/day)	26.4 (24.0-28.2)^ §^	24.5 (23.7-26.8)	24.6 (21.4-25.9)	23.5 (20.5-25.4)	0.015 ^b)^
Protein intake (kcal/IBW/day)	1.1 (1.0-1.2)	1.0 (0.9-1.1)	1.0 (0.8-1.1)	0.9 (0.8-1.1)	0.175 ^b)^

The nutritional characteristics based on GLIM components and the nutritional intakes of the residents among IDDSI framework levels are presented in Table 2. A significant difference in the prevalence of the MUST (1<), low BMI, reduced muscle mass, and reduced food intake or assimilation was noted between IDDSI framework levels 4 and 7. Moreover, residents in IDDSI framework level 4 had significantly lower energy intake (kcal/ideal body weight/day) than those in level 7.

**Figure 3:**
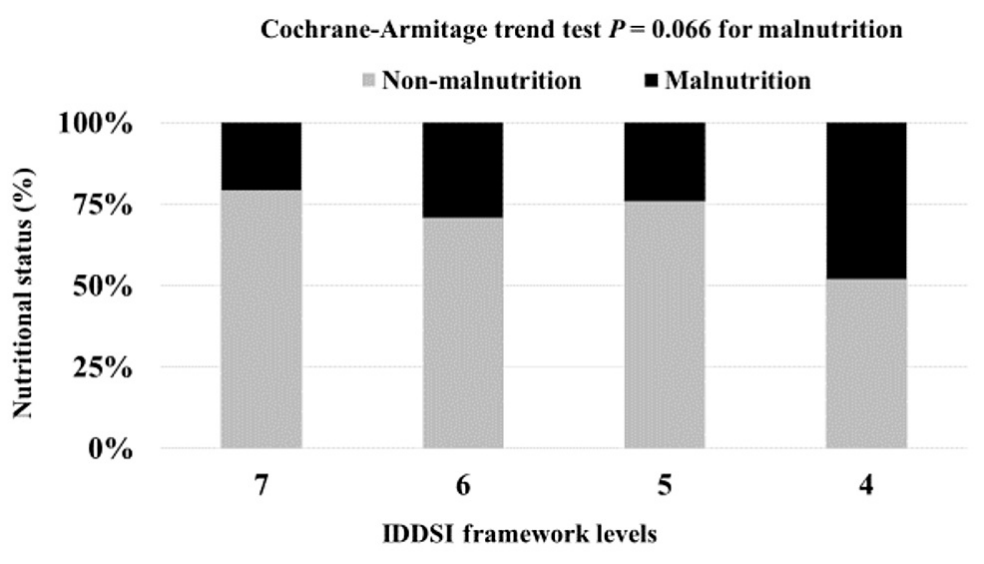
Association between food textures and the nutritional status of residents IDDSI framework levels representing level 4 (pureed), level 5 (minced and moist), level 6 (soft and bite-sized), and level 7 (regular/easy to chew) IDDSI: International Dysphagia Diet Standardization Initiative

The results of the trend analysis of the consumption of food texture levels and the prevalence of malnutrition are shown in Figure 3. The consumption of lower food texture levels was not associated with a higher malnutrition prevalence (p=0.066).

The results of the multivariate logistic regression analysis for malnutrition following simultaneous adjustment for potential confounders are presented in Table 3. After adjusting for the propensity score for covariates, IDDSI level 4 (odds ratio, 5.074; 95% confidence interval, 1.059-28.092; p=0.042) consumption was independently associated with malnutrition.

**Table 3:** Multivariate logistic regression analysis for the presence of GLIM-defined malnutrition R2＝0.269, p≦0.001 The outcome is adjusted for the propensity score. Propensity scores for age, sex, primary diseases for IFMLC admission, CCI, and BI GLIM: Global Leadership Initiative on Malnutrition, CI: confidence interval, IDDSI: International Dysphagia Diet Standardization Initiative, IFMLC: Integrated Facilities for Medical and Long-term Care, CCI: Charlson Comorbidity Index, BI: Barthel Index

Factor	Odds ratio	95% CI	p-value
IDDSI framework levels			
Level 7	Reference	－	－
Level 6	1.678	0.446-6.759	0.446
Level 5	1.541	0.340-7.335	0.574
Level 4	5.047	1.059-28.092	0.042

## Discussion

This study is the first to evaluate the association between food texture levels, as categorized using the IDDSI framework, and malnutrition assessed using the GLIM criteria in IFMLC residents. Malnutrition prevalence was higher among residents who consumed food with lower texture levels. Therefore, consuming a diet with these lower texture levels may be an independent risk factor for malnutrition.

Among IFMLC residents, lower texture levels classified using the IDDSI framework were associated with a higher malnutrition prevalence. Our results further strengthen the validity of previous studies. A systematic review conducted in 2021 examined the relationship between food texture levels and malnutrition, reporting that TMD consumption was correlated with weight loss or malnutrition [23]. Eleven studies in this review investigated the association between TMD intake and malnutrition prevalence, with seven focusing on LTCFs. However, these seven studies did not use the GLIM criteria for nutritional status assessment, which is recommended for evaluating adults with dysphagia [24]. The findings of the present study suggest that the implications of previous research are also applicable to IFMLCs, a new long-term care setting in Japan. In the present study, level 4 (pureed) categorized using the IDDSI framework had a significantly higher prevalence of low BMI, reduced muscle mass, and reduced food intake or assimilation than level 7 (regular). Moreover, the FILS scores in level 4 were significantly lower than those in level 7. Therefore, the level 4 group may have had dysphagia, which caused reduced food intake or assimilation, resulting in a low BMI and reduced muscle mass, thereby leading to malnutrition. Therefore, the impact of dysphagia should be considered a significant factor regarding why lower texture levels classified using the IDDSI framework are associated with a high malnutrition prevalence.

Residents consuming foods with lower texture levels, as classified by the IDDSI framework, have a lower energy intake than those consuming foods with higher texture levels. The results of this study support the findings of a previous study. According to a systematic review and meta-analysis, compared with regular diets, TMD consumption resulted in a lower nutritional intake, particularly in terms of energy and calcium [25]. TMDs frequently offer lower nutritional intake and fewer nutrients than conventional diets [9-11,26]. Specifically, pureed diets require additional water to achieve the desired consistency, thereby rendering them less nutrient-rich than standard diets [9], unless they are specifically enhanced to increase nutrient concentration. The substandard texture and presentation of TMDs may reduce their attractiveness [27], potentially leading to decreased food and nutrient consumption. In this study, the level 4 group had a significantly lower energy intake than the level 7 group. Furthermore, despite the recognition of the significance of TMDs by residents and staff in LTCFs, several issues obstruct the widespread acceptance of pureed foods. These include appearance, taste, smell, inconsistency in preparation and serving, and a lack of variety [28]. Therefore, various factors, including the need for additional water in pureed diets and other cooking-related concerns, may have led to lower energy intake among individuals consuming diets with lower texture levels compared with those consuming diets with higher texture levels.

Appropriate texture modification, shape modification, and adjustment of the consistency of TMDs may be effective ways to improve malnutrition in IFMLC residents. To improve nutritional status, appropriately textured TMDs are required [29]. In particular, shaped TMDs increased energy and protein intakes compared with traditional cook-fresh TMDs [25]. Furthermore, nutrition intervention by adjusting texture and consistency and nutrition enrichment showed positive effects on weight and mealtime satisfaction [23]. Thus, appropriate texture modification, enhancement of the shape, and adjusting the consistency of TMDs may be necessary for IFMFC residents.

This study had some limitations. First, the small sample size and retrospective single-center design may limit the generalizability of the results. Second, as this was a cross-sectional study, the causal relationship between food texture levels and malnutrition remained unclear. Lastly, we did not evaluate the interventions provided for the residents with malnutrition, which may affect their nutritional status.

## Conclusions

The consumption of lower food texture levels categorized using the IDDSI framework was associated with a higher malnutrition prevalence in IFMLC residents. This finding highlights the significance of closely monitoring the food texture levels of TMDs in IFMLC residents. A multicenter cohort study in IFMLCs is necessary to clarify the causal relationship between food texture levels and nutritional status.
